# Lycium barbarum glycopeptide confers neuroprotection in chronic ocular hypertensive rats: a study investigating visual function, retinal structure, microcirculation, and pyroptosis

**DOI:** 10.3389/fphar.2026.1829758

**Published:** 2026-04-30

**Authors:** Chunlin Zhao, Wei Shi, Xuejing Lu

**Affiliations:** 1 Eye School of Chengdu University of TCM, Chengdu, China; 2 Sichuan Provincial Key Laboratory of Traditional Chinese Medicine for Ophthalmic Health, Chengdu, China; 3 Retinal Image Technology and Chronic Vascular Disease Prevention & Control and Collaborative Innovation Center, Chengdu, China; 4 Department of Ophthalmology, Hospital Affiliated of Nanjing University of Chinese Medicine, Nanjing, China

**Keywords:** chronic ocular hypertension, Lycium barbarum glycopeptide, microcirculation, pyroptosis, retinal structure, visual function

## Abstract

**Background:**

Lycium barbarum glycopeptide (LbGp) has been reported to exert neuroprotective effects, yet its role in chronic ocular hypertension (COH) and the underlying mechanisms remain unclear. This study investigated whether LbGp protects against COH-induced retinal damage by inhibiting pyroptosis.

**Methods:**

A COH rat model was established, and the animals were randomly assigned to model, LbGp, VX-765 (pyroptosis inhibitor), and VX-765+LbGp groups. After 28 days of treatment, visual function, retinal structure, microcirculation, and pyroptosis-related protein expression were assessed. In parallel, R28 cells subjected to oxygen-glucose deprivation/reperfusion (OGD/R) were used for *in vitro* validation.

**Results:**

COH model rats exhibited marked visual dysfunction, retinal thinning, microvascular impairment, and upregulation of pyroptosis markers. LbGp treatment significantly attenuated these abnormalities and suppressed pyroptosis activation, showing efficacy comparable to that of VX-765. Notably, combined administration of LbGp and VX-765 produced synergistic effects. *In vitro*, LbGp similarly reduced pyroptosis-related protein expression in OGD/R-exposed R28 cells.

**Conclusion:**

These findings demonstrate that LbGp confers neuroprotection against COH-induced optic nerve injury, at least in part, through inhibition of the pyroptosis pathway. This study provides experimental evidence supporting LbGp as a potential therapeutic candidate for glaucoma.

## 1 Introduction

Glaucoma is an optic neuropathy defined by the progressive loss of retinal ganglion cells (RGCs) and their axons, representing a major cause of irreversible blindness worldwide (Kang and Tanna, 2021). While elevated intraocular pressure (IOP) is widely recognized as a primary risk factor (Jonas et al., 2017), the molecular mechanisms behind optic nerve damage are still not fully understood. This incomplete understanding has, to some extent, hindered the development of neuroprotective therapies. Emerging evidence indicates that pyroptosis significantly contributes to the pathogenesis of multiple neurodegenerative disorders, glaucoma included (Chen et al., 2024; Sun Y. et al., 2024; Qi et al., 2025). Pyroptosis is a form of programmed cell death that depends on the pore-forming activity of gasdermin D (GSDMD) and is typically accompanied by a strong release of pro-inflammatory mediators (Broz et al., 2020; Bai et al., 2025). High IOP accelerates the loss of RGCs and triggers a significant inflammatory cascade in the retina, thereby driving disease progression (Chen et al., 2020; Zeng et al., 2023; Zhang et al., 2021). Therefore, targeting retinal pyroptosis may offer a potential therapeutic approach for reducing neurodegeneration in glaucoma.

Given this, natural products have increasingly become a focus in the search for well-tolerated and effective neuroprotective therapies. *Lycium barbarum L*. (LB), a traditional Chinese herb used both as food and medicine, contains an active component known as lycium barbarum glycopeptide (LbGp). LbGp has been shown to possess anti-inflammatory, antioxidant, and neuroprotective properties (Ren et al., 2024; Zhao et al., 2025; Xu et al., 2024a). In models of neurodegenerative diseases such as Parkinson’s and Alzheimer’s (Lin et al., 2025), in vascular-neural injury models like cerebral ischemia (Xu et al., 2024a; Zhang et al., 2025), and in ocular disease models including vascular-related glaucoma and retinal ischemia-reperfusion injury (RIRI) (Zhao et al., 2025; Lakshmanan et al., 2024; Wu Y. X. et al., 2024; Ou et al., 2025), LbGp has consistently demonstrated significant efficacy. However, in models of chronic ocular hypertension (COH), whether LbGp can protect visual function and whether its mechanism is related to regulating pyroptosis still requires further investigation.

Accordingly, we evaluated the efficacy of LbGp by employing a COH rat model, systematically analyzing its protective effects on visual function, retinal structure, and microcirculation. Furthermore, it explored the impact of LbGp on the molecular-level expression of key pyroptosis execution proteins, including N-GSDMD, and their downstream inflammatory factors, particularly interleukin-1β (IL-1β). This study is expected to clarify the potential mechanism by which LbGp exerts an optic nerve protective effect under COH conditions through inhibiting the pyroptosis pathway, thereby providing new experimental evidence and theoretical support for its development as an auxiliary neuroprotective agent for glaucoma.

## 2 Materials and methods

### 2.1 Animal rearing and experimental grouping

Male SD rats (8–10 weeks old) were acquired from Spivey Biotechnology Co. (Animal Certification No. SCXK (Beijing) 2019-0010) and maintained under standard conditions in the Sichuan Provincial Key Laboratory of Ophthalmic Disease Prevention and Visual Function Protection. All animal experiments were conducted in accordance with the National Standards of the People’s Republic of China (GB/T 35892-2018) and the institutional guidelines for the care and use of laboratory animals. The study protocol was approved by the Animal Ethics Committee of Chengdu University of Traditional Chinese Medicine (Approval No. SYXK 2019-0049).

COH rat was induced by episcleral vein cauterization (EVC) (Bai et al., 2014; Nakamura et al., 2024). Surgical anesthesia was achieved through intraperitoneal injection using 1.0% sodium pentobarbital (30 mg/kg). The rat was anesthetized topically on the surgical eye with 0.4% oxybuprocaine hydrochloride (Benoxil; Santen Pharmaceutical Co., Ltd., Osaka, Japan; Cat. No.: HJ20215002), placed under a surgical microscope, and the eyeball was exposed. An incision was made through the conjunctiva 1 mm posterior to the limbus, followed by blunt dissection to adequately expose the episcleral veins. Three major veins were selectively cauterized using a fine electrocautery device. Successful vascular occlusion was confirmed by proximal vessel dilation and complete cessation of distal blood flow. The sham-operated control animals underwent the same surgical exposure of the veins, but cauterization was deliberately omitted. To prevent infection, the eyes were treated postoperatively with 0.3% tobramycin ointment (Alcon, Houston, TX, United States; Cat. No.: HJ20241126). The model was considered successfully established if the IOP of the experimental eye consistently measured ≥21 mmHg (1 mmHg = 0.133 kPa).

The rats were randomly assigned to either a control group (n = 15) or a model group (n = 60). Control rats received sham surgery on the right eye, while model rats underwent EVC surgery on the right eye. The untreated contralateral eye in each animal served as an internal control. After successful model establishment, the model group rats were randomly allocated to one of the four experimental groups (n = 15 per group): Model group (daily intraperitoneal injection of saline combined with oral gavage of saline), LbGp group (daily intraperitoneal injection of saline combined with oral gavage of LbGp at 100 mg/kg), VX-765 group (daily intraperitoneal injection of VX-765 at 50 mg/kg combined with oral gavage of saline), and the combination treatment (VX-765 + LbGp) group (daily intraperitoneal injection of VX-765 at 50 mg/kg combined with oral gavage of LbGp at 100 mg/kg). VX-765 was purchased from MedChemExpress (United States, Cat. No.: HY-13205) (Dong et al., 2022), and LbGp was purchased from Tianren Gougou Biotechnology Co., Ltd. (Ningxia, China, Cat. No.: 20241218) (Zhao et al., 2025). Both intraperitoneal injection and oral gavage were lasted for 28 consecutive days in all groups (Lakshmanan et al., 2024). Following the intervention period, a series of tests were sequentially performed, including the IOP measurement, optomotor response test (ORT), multifocal electroretinography (mfERG), routine ERG, flash visual evoked potentials (f-VEP), and optical coherence tomography/angiography (OCT/OCTA). Subsequently, retinal tissues were harvested for hematoxylin and eosin (HE) staining and Western blot (WB) analysis.

### 2.2 Cell culture and treatment grouping

R28 cells (Kerafast, Boston, MA, United States) were cultured in DMEM (Solarbio, Beijing, China; Cat: 31600) supplemented with 10% FBS (Zunyan Bio-technology Co., Ltd., China; Cat: HBT0100236) and 1% penicillin-streptomycin (P/S, Solarbi, Beijing, China; Cat: P1400). Cells were kept under a controlled atmosphere of 5% CO_2_ at a constant temperature of 37 °C.

Cells were seeded into two experimental groups: a control and a model group. Control cells were cultured under normoxic conditions (5% CO_2_ and 95% O_2_) in complete medium for 24 h. Model cells were first incubated for 4 h in glucose- and serum-free medium under hypoxic conditions (5% CO_2_ and 95% N_2_), followed by replacement with complete medium and incubation under normoxic conditions for 20 h to induce the oxygen-glucose deprivation/reoxygenation (OGD/R) model. Subsequently, the cells were distributed into four experimental groups: model, LbGp, VX-765, and VX-765 + LbGp. All groups underwent the 24 h OGD/R modeling procedure. During the intervention, the LbGp group was treated with 1,000 ng/mL LbGp in complete medium, the VX-765 group received 10 μmol/L VX-765 in complete medium, and the VX-765 + LbGp group was administered both 1,000 ng/mL LbGp and 10 μmol/L VX-765 in complete medium (Xu et al., 2024a; Sun et al., 2020). All experimental conditions were independently repeated three times, and within each replicate, three technical replicate wells were set for each treatment group. After the experiment, cells from each group were collected for Hoechst 33342/PI staining.

### 2.3 ORT

After 28 days of intervention, ORT was performed with the OptoTrack system (Shanghai Xinruan Information Technology Co., Ltd., Shanghai, China), which automatically recorded and analyzed the optokinetic response index (OKR) index and head rotation speed. Rats were placed on a central platform within a four-screen arena displaying moving vertical sine-wave gratings. The stimuli, presented at a constant speed (12°/s) and 100% contrast, spanned six spatial frequencies (0.02, 0.1, 0.15, 0.2, 0.3, and 0.4 cycles/degree) to determine the tracking threshold. To reduce random error, each frequency was tested eight times in a randomized sequence per session. All experiments were conducted by the same technician, and image analysis was performed by the same physician.

### 2.4 mfERG and routine ERG

After 28 days of intervention, mfERG was performed using the RETISCAN 3.15 visual electrophysiology system (Roland Consult, Brandenburg, Germany). Following a 12-h overnight dark adaptation period, rats were anesthetized via intraperitoneal administration of 1% pentobarbital sodium (30 mg/kg body weight). Pupils were then fully dilated with 1% tropicamide eye drops (Yongguang Pharmaceutical Co., Ltd., Hebei, China; Cat. No. 0042218) prior to testing. After rats immobilization, the recording electrode was positioned at the corneal limbus while the reference and ground electrodes were subcutaneously positioned on the forehead and tail, respectively. Once electrode impedance met the requirements, the program was initiated to record the amplitude and implicit time. For routine ERG testing, the Diagnosys visual electrophysiology system (Diagnosys, United States; model: Celeris-D430) was used. Anesthesia and pupil dilation procedures were consistent with those described above. Prior to recording, the cornea was kept moist using viscoelastic agent Aiwei (Bausch and Lomb, United States; Cat. No. 01322). Electrodes were placed at standard sites: the cornea (active electrode), nose (reference), head (active), and tail (ground). Signal acquisition began once impedance was confirmed. To reduce inter-operator variability, all recordings were performed by the same technician, and all analyses were conducted by the same physician.

### 2.5 VEP

After 28 days of intervention, f-VEP recordings were conducted using both the OPTO-ERG (Beijing HealthOLight Technology) and Diagnosys electrophysiology systems. All recordings were performed under standardized conditions of dark adaptation, anesthesia, and pupil dilation. Electrode placement followed each device’s protocol: the OPTO-ERG system used subcutaneous occipital (active), cheek (reference), and tail (ground) electrodes, while the Diagnosys system applied the previously described configuration. Signal acquisition began once impedance was verified. A single technician performed all recordings, and the data were consistently analyzed by one physician to ensure procedural uniformity.

### 2.6 OCT/OCTA

After 28 days of intervention, OCT/OCTA examination was performed. Anesthesia and pupil dilation were conducted as previously described. Rats were placed on the animal platform, and imaging was performed using a TowardPi device (TowardPi Medical Technology, Beijing, China; model: BM-400K). Following image acquisition, the TowardPi software was used to analyze the thickness of the retinal nerve fiber layer (RNFL), ganglion cell complex (GCC), and full retinal thickness, along with assessing the superficial, deep, and total vascular densities (SVD, DVD, TVD). All images were acquired by the same technician and analyzed by the same physician.

### 2.7 HE

Retinal tissues were dissected, dehydrated through a graded ethanol series, cleared, and subsequently embedded in paraffin. Consecutive sections were obtained from the paraffin blocks using a Leica microtome (Leica, Shanghai, China; model: RM2016) to obtain tissue slices for subsequent analysis. Following dewaxing with xylene and rehydration via an ethanol gradient, the sections were stained following the protocol provided with the HE staining kit (Beyotime Biotechnology, Shanghai, China; Cat. No. C0105S): staining with hematoxylin, followed by eosin staining, then gradient dehydration in absolute ethanol, clearing in xylene, and finally mounting with neutral resin. Images were acquired with a Nikon optical microscope (Nikon, Japan; model: Eclipse E100) and quantitatively analyzed with ImageJ software to evaluate pathological changes in retinal tissues. All procedures were performed under standard laboratory conditions to ensure the reliability of the experimental results.

### 2.8 Hoechst 33342/PI

R28 were cultured according to the designated groups, and nuclear and viability staining was carried out using the Hoechst 33342/PI dual-staining kit (Beyotime Biotechnology, Shanghai, China; Cat: P0137). Staining buffer, Hoechst 33342, and PI staining solutions were added to each group of R28s and incubated at 4 °C under light-protected conditions for 20 min. Following PBS washes, the cells were visualized under a fluorescence microscope (Nikon Corporation, Tokyo, Japan; machine model: Eclipse C1). Image analysis was performed using ImageJ software.

### 2.9 WB

Protein extraction was performed with RIPA lysis buffer (Servicebio, Wuhan, China; Cat: G2002) along with the protease phosphatase inhibitor mixture (Beyotime, Shanghai, China; Cat: P1045). The extracted proteins were separated via SDS-PAGE and subsequently transferred onto PVDF membranes. Membranes were blocked with QuickBlock™ Protein-Free WB Solution (Beyotime, Shanghai, China; Cat: P0240) for 20 min and incubated with primary antibodies overnight. Following three 10-min washes with TBST, the membranes were incubated with species-matched secondary antibodies for 2 h. Following three further TBST washes, protein bands were detected using an electrochemiluminescence (ECL) kit (Beyotime, Shanghai, China; Cat: P0018S) and visualized using a Chemiluminescence Imaging System. ImageJ software was used to analyze protein band intensity.

The following primary antibodies were used: pro-caspase-1 (1:1,000, Abcam, Cambridge, United Kingdom; Cat: ab179515), caspase-1 (1:1,000, ABclonal, Woburn, MA, United States; Cat: A23429), N-GSDMD (1:1,000, ABclonal, Woburn, MA, United States; Cat: A24059), GSDMD (1:1,000, ABclonal, Woburn, MA, United States; Cat: A24476), Pro-IL-1β (1:1,000, ABclonal, Woburn, MA, United States; Cat: A1112), IL-1β (1:1,000, ABclonal, Woburn, MA, United States; Cat: A23484), and β-actin (1:1,000, Servicebio, Wuhan, China; Cat: GB15003). Secondary antibodies used were HRP-goat anti-rabbit IgG (1:8,000, Servicebio, Wuhan, China; Cat: GB23303) and HRP-goat anti-mouse IgG (1:5,000, Servicebio, Wuhan, China; Cat: GB25301).

### 2.10 Statistical analysis

Statistical analyses were performed using SPSS 26.0 (IBM, Armonk, NY, United States) and GraphPad Prism 8.1 (La Jolla, CA, United States). Between-group comparisons were performed using the t-test, whereas multivariate analyses were conducted with one-way or two-way ANOVA. Data were presented as mean ± standard deviation (SD), with statistical significance defined as P < 0.05.

## 3 Results

### 3.1 IOP profiles of each group

**FIGURE 1:**
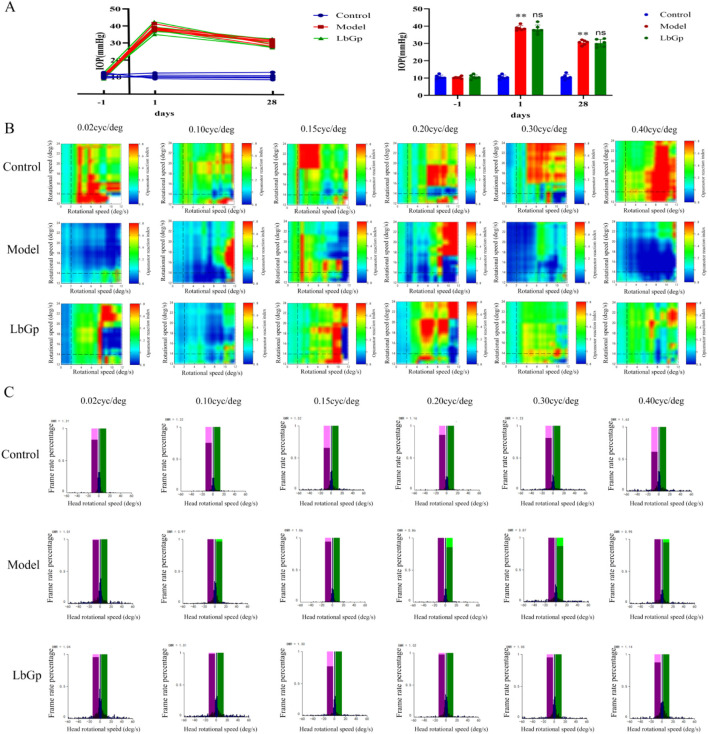
IOP profiles of each group and changes in rat ORT across groups. **(A)** IOP measurements before modeling (day -1), on day 1 after modeling, and after 28 days of intervention. The data are presented as mean ± SD (n = 6 per group). Blue represent Control group, red represent Model group, and green represent LbGp group. p-values: **<0.01 vs. Control; ns > 0.05 vs. Model. **(B)** Heat maps of ORT at different spatial frequencies of rats. A red-blue continuous gradient is used: colors closer to red indicate higher sensitivity, colors closer to blue indicate lower sensitivity, and intermediate colors (e.g., yellow, green) indicate moderate responses. **(C)** Histogram of head rotation speed of rats at different spatial frequencies. Purple and green represent syndirectional and contra-directional rotations, respectively. At each spatial frequency, OMR in the model group was reduced compared to the control group; OMR in the treatment group was improved at each frequency compared to the model group.

Before modeling (day -1), the IOP values in the three groups were 10.58 ± 1.12, 10.28 ± 0.59, and 10.58 ± 1.19 mmHg, respectively, with no significant difference among the groups (p > 0.05). On day 1 after modeling, the IOP values in the three groups were 10.53 ± 1.14, 38.82 ± 1.39, and 38.17 ± 2.54 mmHg, respectively. Compared with the control group, the IOP in both the model group and the LbGp group was significantly increased (both p < 0.01), but there was no significant difference between these two groups (p > 0.05). After 28 days of LbGp intervention, the IOP values in the three groups were 10.62 ± 1.45, 30.48 ± 1.36, and 30.17 ± 2.16 mmHg, respectively. The IOP in the model group and the treatment group remained significantly higher than that in the control group (both p < 0.01), and there was still no significant difference between the two groups (p > 0.05). Refer to Figure 1A. These IOP data indicate that EVC can induce and maintain elevated IOP for at least 28 days; LbGp has no significant IOP-lowering effect; and the IOP differences within each group are minimal, thus excluding the interference of IOP variation on other outcome measures.

### 3.2 LbGp preserves visual function in COH rats

ORT testing revealed that control group rats were highly responsive, showing elevated OKR values accompanied by rapid head-tracking movements. Both OKR values and head rotation speed were significantly reduced in the model group compared to controls, indicating substantial impairment of the visuomotor pathway. LbGp treatment significantly improved both OKR and head rotation speed relative to the untreated model group, yet the values remained below control levels. Refer to Figures 1B,C. These findings suggest that LbGp partially restores the function of the visuomotor pathway.

**FIGURE 2:**
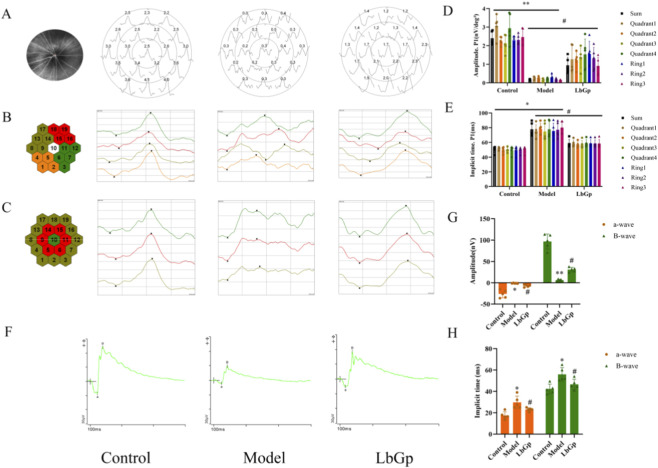
Results of mfERG and routine ERG in each group of rats. **(A)** The typical mfERG waveforms of each groups. The diagram shows first-order kernel waveforms recorded from 19 hexagonal areas of a single rat retina. The x-axis of each waveform within each hexagon represents time (ms), and the y-axis represents amplitude (nV/deg^2^). All waveforms display a characteristic negative-positive biphasic configuration (N1 and P1 waves). **(B)** Representative images are shown for each of the four retinal quadrants: superior temporal, inferior temporal, superior nasal, and inferior nasal. **(C)** Representative images across the three concentric recording rings are displayed: central, mid-peripheral, and peripheral. **(D,E)** Statistical analysis of mfERG amplitudes and implicit time. The data are presented as mean ± SD (n = 5 per group). p-values: *<0.05 vs. Control, **<0.01 vs. Control; #<0.05 vs. Model. **(F)** The typical routine ERG waveforms of each groups. **(G,H)** Statistical analysis of routine ERG amplitudes and implicit time. The data are presented as mean ± SD (n = 5 per group). p-values: *<0.05 vs. Control, **<0.01 vs. Control; #<0.05 vs. Model.

The mfERG results showed that compared with the control group (2.40 ± 0.37 nV), the P1-wave amplitude in the model group was significantly reduced to 0.17 ± 0.03 nV (p < 0.01), while the implicit time was markedly prolonged from 50.32 ± 4.29 ms in the control group to 77.84 ± 9.25 ms (p < 0.05), indicating impaired retinal neuronal signal conduction. After LbGp intervention, both parameters improved: the P1-wave amplitude recovered to 0.94 ± 0.43 nV (p < 0.05 vs. model group), and the implicit time shortened to 58.70 ± 5.51 ms (p < 0.05 vs. model group), suggesting that LbGp exerts a restorative effect on retinal neural signaling function. Refer to Figures 2A–E.

The routine ERG results revealed that, compared with the control group (a-wave amplitude: 26.19 ± 9.06 nV; b-wave amplitude: 96.67 ± 17.45 nV; a-wave implicit time: 17.40 ± 3.52 ms; b-wave implicit time: 42.20 ± 4.58 ms), the model group exhibited a significant reduction in a-wave amplitude to 1.16 ± 0.64 nV (p < 0.05) and in b-wave amplitude to 5.89 ± 2.53 nV (p < 0.01), along with a prolonged a-wave implicit time of 29.70 ± 5.93 ms (p < 0.05) and a prolonged b-wave implicit time of 55.90 ± 6.31 ms (p < 0.05), indicating impairment of the signaling pathway from photoreceptors to the inner retina. Following LbGp intervention, the treated group showed recovery in these parameters: a-wave amplitude increased to 6.95 ± 3.03 nV (p < 0.05 vs. model group), b-wave amplitude to 31.04 ± 5.39 nV (p < 0.05), a-wave implicit time shortened to 23.00 ± 1.62 ms (p < 0.05), and b-waveimplicit time to 46.50 ± 4.87 ms (p < 0.05), suggesting that the intervention promotes repair of the retinal neural pathway. Refer to Figures 2F,G.

**FIGURE 3:**
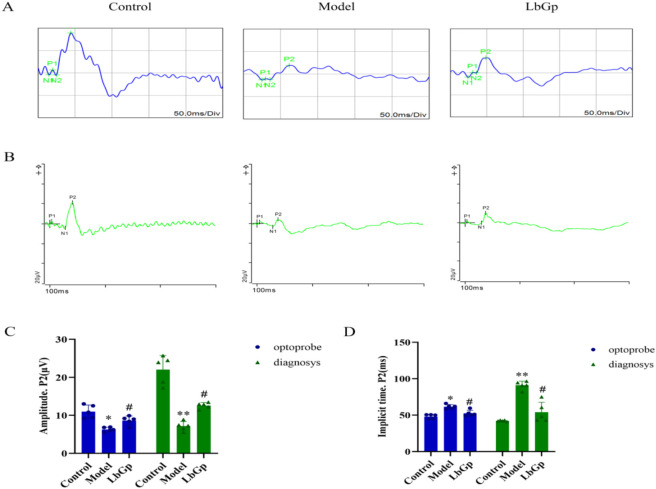
Results of f-VEPs in each group of rats. **(A,B)** The typical f-VEPs waveforms of each groups. The x-axis represents time (ms), and the y-axis represents amplitude (μV). The waveform diagram is annotated with the classic waveform components, including N1, P1, P2, etc. The blue waveform in A is detected by optoprobe system. The green waveform in B is detected by diagnosys system. **(C,D)** Statistical analysis of the amplitudes and implicit time. The data are presented as mean ± SD (n = 5 per group). p-values: *<0.05 vs. Control, **<0.01 vs. Control; #<0.05 vs. Model.

The f-VEPs results showed that under both testing conditions, the P2-wave amplitude in the model group was significantly reduced. In Test 1, it decreased from 10.98 ± 1.74 μV in the control group to 6.28 ± 0.58 μV (p < 0.05), and in Test 2, from 22.02 ± 3.76 μV to 7.15 ± 1.23 μV (p < 0.01). Concurrently, the P2-wave implicit time was consistently prolonged, increasing from 48.10 ± 3.07 ms in the control group to 61.40 ± 3.13 ms in Test 1 (p < 0.05), and from 42.20 ± 0.84 ms to 91.00 ± 5.56 ms in Test 2 (p < 0.01), indicating functional impairment along the visual pathway, particularly from the optic nerve to the visual cortex. After LbGp intervention, these abnormal changes were improved. In Test 1, the P2-wave amplitude recovered to 8.64 ± 1.21 μV (p < 0.05 vs. model group), and the latency shortened to 52.50 ± 4.11 ms (p < 0.05). In Test 2, the amplitude increased to 12.58 ± 0.79 μV (p < 0.05), and the implicit time decreased to 53.90 ± 13.79 ms (p < 0.05). These results suggest that LbGp has a beneficial effect on visual pathway conduction and neural synchrony. Refer to Figure 3.

### 3.3 LbGp improves retinal structure in COH rats

**FIGURE 4:**
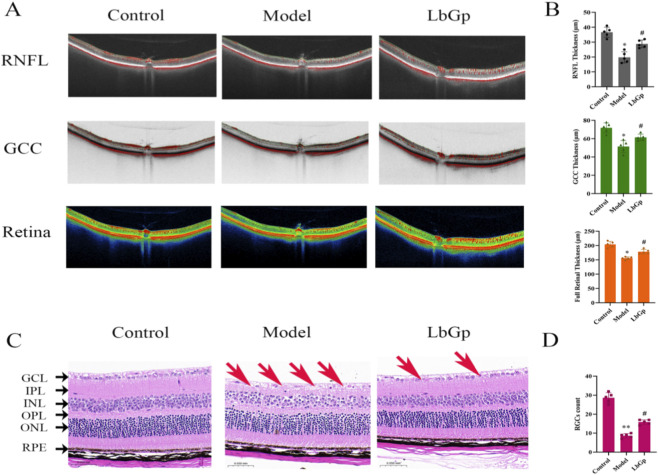
Results of retinal structure in each group of rats. **(A)** Retinal OCT test of rats. OCT was performed to image the retinas of rats in each group, obtaining cross-sectional structural images of the retina. The figure clearly shows the layered structures of the retina, which can be used to assess the thickness of each retinal layer. Scale = 200 μm. **(B)** Statistical analysis of the thickness of RNFL, GCC, and full retina. The data are presented as mean ± SD (n = 5 per group). p-values: *<0.05 vs. Control; #<0.05 vs. Model. **(C)** Retinal HE staining of rats. Scale = 50 μm. RGCs are denoted by red arrows. **(D)** Statistical analysis of RGCs Counts. The data are presented as mean ± SD (n = 5 per group). p-values: **<0.01 vs. Control; #<0.05 vs. Model.

OCT results showed that in the control group, the RNFL thickness, GCC thickness, and full retinal thickness were (37.00 ± 3.16) μm, (71.50 ± 4.93) μm, and (204.50 ± 9.65) μm, respectively. Compared with the control group, the model group exhibited significantly reduced RNFL thickness (20.67 ± 3.93 μm), GCC thickness (51.83 ± 5.98 μm), and full retinal thickness (157.50 ± 6.09 μm) (all p < 0.05). Following drug intervention, the treated group demonstrated increased RNFL thickness (29.00 ± 2.37 μm), GCC thickness (61.83 ± 3.31 μm), and full retinal thickness (176.83 ± 8.42 μm), all of which were markedly higher than the corresponding measurements in the model animals (p < 0.05). Refer to Figures 4A,B.

HE staining revealed well-organized retinal layers and intact cytoarchitecture in the control group, with RGCs count of 28.60 ± 2.70 cells per field. In contrast, retinal sections from the model group showed evident structural disorganization and a significant reduction in RGCs density to 8.60 ± 1.14 cells per field (p < 0.01). Treatment with LbGp notably ameliorated these pathological alterations, resulting in better-preserved retinal layering and an increased RGCs count of 15.80 ± 1.30 cells per field, which was markedly more than the corresponding measurements in the model animals (p < 0.05). Refer to Figures 4C,D.

### 3.4 LbGp ameliorates retinal microcirculation in COH rats

**FIGURE 5:**
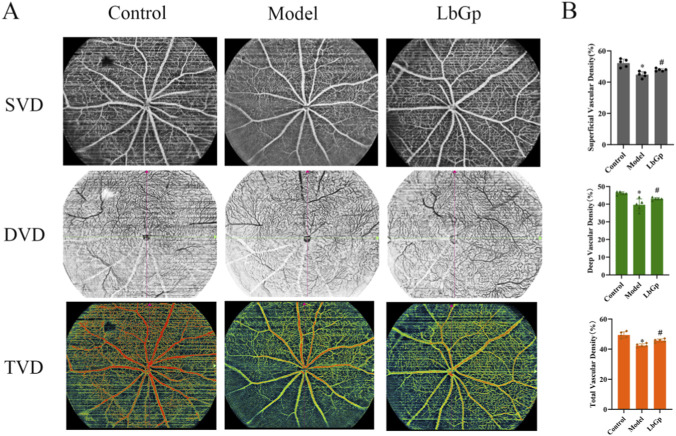
Results of retinal microcirculation in each group of rats. **(A)** Representative blood flow imaging images of the SVD, DVD, and TVD in the retina of rats in each group. The figure shows the distribution of blood flow signals in the retinal vascular layers of different experimental groups. Scale = 200 μm. **(B)** Statistical analysis of the vascular density of SVD, DVD, and TVD. The data are presented as mean ± SD (n = 5 per group). p-values: *<0.05 vs. Control; #<0.05 vs. Model.

OCTA results showed that in the control group, SVD was (52.33 ± 2.34)%, DVD was (46.67 ± 1.37)%, and TVD was (49.00 ± 2.10)%. Compared with the control group, the model group exhibited significant reductions in SVD, DVD, and TVD to (44.83 ± 1.72)%, (39.83 ± 3.06)%, and (41.83 ± 1.72)%, respectively (all p < 0.05). Following drug intervention, the treated group demonstrated increases in SVD, DVD, and TVD to (48.00 ± 0.89)%, (43.33 ± 1.03)%, and (45.50 ± 1.05)%, respectively, all of which were markedly higher than the corresponding measurements in the model animals (p < 0.05). Refer to Figure 5.

### 3.5 Association between COH rats and pyroptosis

**FIGURE 6:**
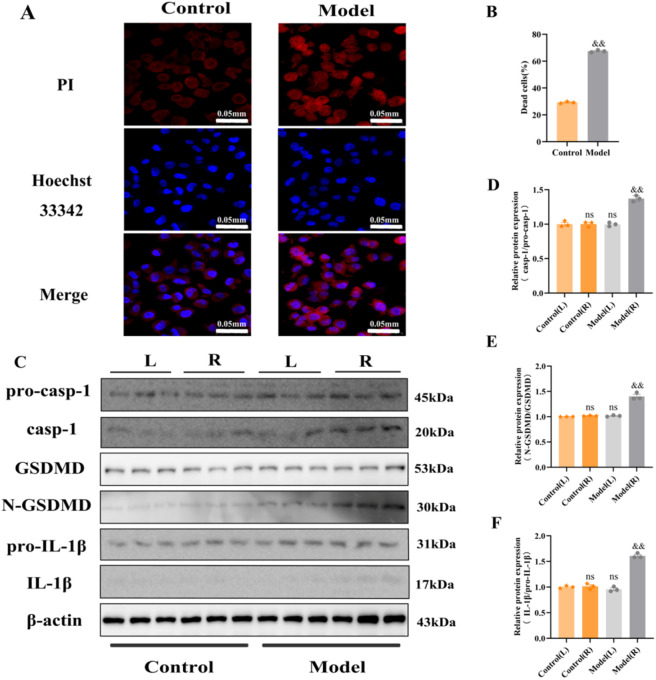
Model group shows pyroptosis. **(A)** Representative micrographs depicting Hoechst 33342/PI co-staining are presented across all groups. Red staining and blue staining indicate PI (dead cells) and Hoechst 33342 (live cells), respectively. Scale bar = 0.05 mm. **(B)** Quantification of dead cells across experimental groups. The data are presented as mean ± SD (n = 3 independent experiments). p-values: && < 0.01 vs. Control. **(C)** WB analysis shows the retinal expression levels of key pyroptosis-related proteins, including pro-caspase-1, caspase-1, GSDMD, N-GSDMD, pro-IL-1β, and IL-1β, across all experimental groups. **(D–F)** Quantitative analysis of pro-caspase-1, caspase-1, GSDMD, N-GSDMD, pro-IL-1β, and IL-1β proteins in each group. The data are presented as mean ± SD (n = 3 per group). p-values: ns > 0.05 vs. Control, && < 0.01 vs. Model(L).

Hoechst 33342/PI staining revealed minimal red fluorescence in control cells, whereas the model group showed a marked increase in signal intensity (p < 0.01). In this assay, red fluorescence (PI) marks dead cells and blue fluorescence (Hoechst 33342) marks live cells. Refer to Figures 6A,B. To validate the cell staining results, we performed WB analysis on animal retinal tissue. The results showed that key proteins associated with pyroptosis, including caspase-1, N-GSDMD, and IL-1β, were barely detectable in the left eyes (sham-operated self control eyes) of the control group, the right eyes (sham-operated eyes) of the control group, and the left eyes (operated self control eyes) of the model group. No statistically significant differences were observed among these three groups (p > 0.05). In contrast, the expression of caspase-1, N-GSDMD, and IL-1β was significantly upregulated in the right eyes (operated eyes) of the model group (p < 0.01). Refer to Figures 6C–F. These findings indicate that COH conditions can induce pyroptosis in the eye.

### 3.6 LbGp exerts neuroprotective effects by suppressing pyroptosis

**FIGURE 7:**
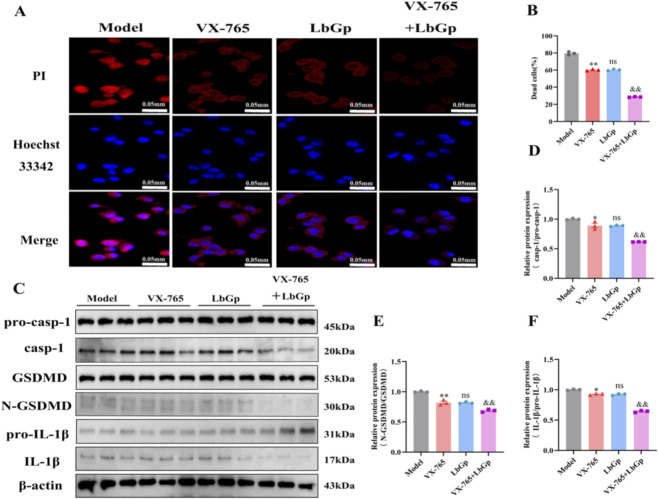
LbGp exerts anti-pyroptotic effects. **(A)** Representative micrographs depicting Hoechst 33342/PI co-staining are presented across all groups. Red staining and blue staining indicate PI (dead cells) and Hoechst 33342 (live cells), respectively. Scale bar = 0.05 mm. **(B)** Quantification of dead cells across experimental groups. The data are presented as mean ± SD (n = 3 independent experiments). p-values: **< 0.01 vs. Model; ns > 0.05 vs. VX-765; && < 0.01 vs. LbGp. **(C)** WB shows the expression of pro-caspase-1, caspase-1, GSDMD, N-GSDMD, pro-IL-1β, and IL-1β proteins in the retinas of rats from each group. **(D–F)** Quantitative analysis of pro-caspase-1, caspase-1, GSDMD, N-GSDMD, pro-IL-1β, and IL-1β proteins in each group. The data are presented as mean ± SD (n = 3 per group). p-values: *< 0.05 vs. Model, **< 0.01 vs. Model; ns > 0.05 vs. VX-765; && < 0.01 vs. LbGp.

Hoechst 33342/PI staining showed strong red fluorescence in the model group. Compared with the model group, red fluorescence intensity was significantly reduced in both the VX-765 group and the LbGp group (p < 0.01). No statistically significant difference was observed in red fluorescence intensity between the VX-765 group and the LbGp group (p > 0.05). Furthermore, a stronger reduction in red fluorescence intensity was observed in the VX-765 + LbGp group compared to either agent when administered individually (p < 0.01). Refer to Figures 7A,B. To validate the *in vitro* cell staining results, we performed WB analysis on animal retinal tissue. Key pyroptosis-associated proteins were markedly upregulated in the model group, as evidenced by elevated expression of caspase-1, N-GSDMD, and IL-1β. In contrast to the model group, both the VX-765 and LbGp intervention groups demonstrated a marked downregulation in the expression of these pyroptosis-related proteins (p < 0.05, p < 0.01, and p < 0.05, respectively). No statistically significant differences were observed in the expression of these pyroptosis-related proteins between the VX-765 group and the LbGp group (all p > 0.05). Expression levels of caspase-1, N-GSDMD, and IL-1β were notably lower in the combination treatment group relative to monotherapy with VX-765 or LbGp alone (all p < 0.01). Refer to Figures 7C–E. These findings suggest that LbGp can significantly inhibit pyroptosis induced by COH conditions.

## 4 Discussion

By integrating systematic evaluations from three dimensions—visual function and retinal structure, retinal microcirculation, and cellular molecular mechanisms—this study comprehensively delineates the multi-level pathological characteristics of visual impairment in the COH rat model and explores the potential mechanisms through which LbGp exerts neuroprotective effects via multi-level synergistic actions.

LbGp ameliorates COH-induced visual and structural damage. Compared to controls, model rats showed reduced mfERG/ERG amplitudes (P1: 2.40→0.17 nV; a/b: 26.19/96.67→1.16/5.89 nV) and f-VEP P2 amplitudes (Test1: 10.98→6.28 μV; Test2: 22.02→7.15 μV), with prolonged latencies; LbGp significantly improved all parameters (Figures 2, 3). Structural assessments further supported these functional changes. OCT and HE staining (Figure 4) confirmed structural recovery after LbGp treatment. Compared with controls (RNFL: 37.00 ± 3.16 μm, GCC: 71.50 ± 4.93 μm, total retina: 204.50 ± 9.65 μm; RGCs: 28.60 ± 2.70/field), model rats showed significant reductions (RNFL: 20.67 ± 3.93 μm, GCC: 51.83 ± 5.98 μm, total: 157.50 ± 6.09 μm; RGCs: 8.60 ± 1.14/field), while LbGp significantly restored these parameters (RNFL: 29.00 ± 2.37 μm, GCC: 61.83 ± 3.31 μm, total: 176.83 ± 8.42 μm; RGCs: 15.80 ± 1.30/field). Their development is frequently associated with a number of underlying pathological processes, such as oxidative stress, impaired mitochondrial function, and persistent neuroinflammation (Rak et al., 2025; Rayner et al., 2023; Sgambellone et al., 2023; Grotegut et al., 2020; Wu et al., 2019). For example, excessive reactive oxygen species (ROS) can accumulate and harm photoreceptor outer segments, as well as disrupt the metabolism and transmission of neurotransmitters (Zeng et al., 2025; Li et al., 2019). These processes may explain the ERG abnormalities observed in the study. At the same time, the inflammatory microenvironment and hypoxic conditions caused by elevated IOP may contribute to axonal demyelination, neuronal loss, and reduced synaptic efficiency (Villoslada et al., 2019; Parrilla et al., 2024; Liu et al., 2025; Xia et al., 2024; Parisi et al., 2021; Rocha et al., 2020; Jassim et al., 2022; Van Hook, 2022; El et al., 2024). These changes are likely responsible for the observed f-VEP abnormalities. Following treatment with LbGp, all evaluated parameters showed significant improvement, though they did not fully return to baseline levels. This result suggests that LbGp has partial reparative and neuroprotective effects, which are likely mediated through synergistic mechanisms. At the retinal level, LbGp may work through several mechanisms. It could boost antioxidant defenses by activating the nuclear factor erythroid 2-related factor 2 (Nrf2)/heme oxygenase 1 (HO-1) pathway (Yang et al., 2023), reduce inflammation by suppressing the nuclear factor kappa-B (NF-κB) pathway (Zhao et al., 2025; Lakshmanan et al., 2024; Jiang et al., 2023), and promote the expression of neurotrophic factors like brain-derived neurotrophic factor (BDNF) to help RGCs survive (Chen et al., 2025; Xu et al., 2024b). Additionally, it may help maintain proper neuronal excitability and signal transmission efficiency by regulating calcium balance and stabilizing mitochondrial membrane potential (Hu et al., 2018; Kou et al., 2017). At the central level, the benefits of LbGp may partly result from enhanced afferent signal input to the CNS, likely due to improved retinal function. In addition, active components (such as LB extract) may also act directly on the central system. This includes activating the Wnt pathway (Han et al., 2025), promoting a neuroprotective microglial phenotype (Sun Z. et al., 2024), reducing axonal loss in the optic nerve, and preserving myelin structure (Li et al., 2015). Together, these effects support neural plasticity, myelin repair, and improved neural conduction efficiency.

LbGp ameliorated retinal microcirculation impairment induced by the COH model. OCTA results (Figure 5) showed that in the control group, SVD was (52.33 ± 2.34)%, DVD was (46.67 ± 1.37)%, and TVD was (49.00 ± 2.10)%. Compared with the control group, the model group exhibited significantly reduced SVD, DVD, and TVD, which decreased to (44.83 ± 1.72)%, (39.83 ± 3.06)%, and (41.83 ± 1.72)%, respectively (all p < 0.05). Following LbGp intervention, SVD, DVD, and TVD in the treatment group increased to (48.00 ± 0.89)%, (43.33 ± 1.03)%, and (45.50 ± 1.05)%, respectively, all of which were significantly higher than the corresponding measurements in the model group (Bojikian et al., 2019). Importantly, dysfunction in the microcirculation is closely connected to the functional and structural deficits described earlier. Reduced SVD impairs blood flow in the superficial layers of the retina and disrupts the regulatory microenvironment of the RNFL (Qu et al., 2018; Rao et al., 2020). The resulting ischemia and hypoxia may accelerate damage to axons and myelin (Miller et al., 2024; Filippatou et al., 2025), which could help explain the abnormalities observed in f-VEPs and ORT. Meanwhile, a decrease in DVD suggests reduced oxygen and nutrient supply to the inner nuclear layer (INL) and the photoreceptor layer. Inadequate perfusion can worsen oxidative stress and inflammatory responses (Arrigo et al., 2021; Venkatesh et al., 2019; Moon et al., 2018; Fragiotta et al., 2023), thereby increasing damage to photoreceptors and disrupting neurotransmitter regulation (Yang et al., 2022; Wu S. et al., 2024). This mechanism offers a plausible explanation for the ERG abnormalities observed in the study. Collectively, SVD and DVD serve as critical indicators for assessing retinal microvascular function and neurovascular unit integrity (Wu et al., 2023; Wong et al., 2021; Waheed et al., 2023; Wang et al., 2020). Following LbGp intervention, concurrent improvements were observed in microcirculatory, functional, and structural parameters. This coordinated improvement across multiple dimensions suggests an integrated, synergistic mechanism behind its neuroprotective effects. In terms of the underlying microcirculatory mechanisms, previous studies have demonstrated that LB extract can boost antioxidant enzyme expression by activating the kelch-like ECH-associated protein 1 (Keap1)/Nrf2 pathway. It also helps suppress the release of inflammatory factors, such as IL-α and IL-1β, which are mediated by NF-κB, ultimately alleviating vascular endothelial injury (Hu et al., 2021; David et al., 2023; Wu et al., 2012). Additionally, it helps regulate the expression of angiogenesis-related proteins such as vascular endothelial growth factor A (VEGFA), VEGFR2, and angiotensin, which in turn inhibits endothelial cell apoptosis and promotes vascular stabilization and remodeling (Zhu et al., 2022; Xue et al., 2019). These effects not only directly improve microcirculatory perfusion but also indirectly support neuronal survival and signal conduction by restoring adequate oxygen and nutrient supply to the retina. Together, these pathways work in synergy to promote comprehensive recovery of both function and structure.

LbGp inhibits retinal pyroptosis induced by the COH model. PI staining (Figures 6A,B, 7A,B) showed that the proportion of dead cells in the model group was significantly increased compared with the control group, while the LbGp group exhibited a marked reduction compared with the model group. WB results (Figures 6C–F, 7C–F) demonstrated that the protein expression levels of caspase-1, N-GSDMD, and IL-1β in the model group were significantly higher than those in the control group, whereas these protein expression levels were significantly lower in the LbGp group than in the model group. Recent studies have shown that sustained mechanical stress and ischemic-hypoxic conditions can promote the release of ROS. These signals activate the NOD-like receptor thermal protein domain associated protein 3 (NLRP3) inflammasome, triggering pyroptosis through the caspase-1/GSDMD pathway and leading to the release of large amounts of pro-inflammatory factors, including IL-1β. This process intensifies local inflammatory responses (Chen et al., 2020; Zeng et al., 2023; Pronin et al., 2019; Huang et al., 2021). However, inhibiting pyroptosis can effectively reduce inflammation and alleviate the tissue damage. Studies have shown that LB extract inhibits NLRP3 inflammasome activation (Zhou et al., 2022), regulates the Nrf2/HO-1 pathway, and inhibits pyroptosis triggered by hypoxia/reoxygenation, leading to neuroprotective effects (Gao et al., 2024). LbGp can regulate lipid metabolism, suppress the mitogen-activated protein kinase (MAPK)/NF-κB signaling pathway, downregulate key pyroptosis-related proteins, and reduce neuroinflammation (Jiang et al., 2023). Based on current evidence, we proposet that LbGp may inhibit pyroptosis through multiple pathways. Its mechanism likely involves either directly suppressing NLRP3 inflammasome activation or indirectly modulating related signaling cascades, such as the NF-κB pathway. This action reduces RGCs loss while also improving the surrounding inflammatory environment. As a result, it helps achieve coordinated neuroprotective benefits at the functional, structural, and microcirculatory levels.

By using a multimodal approach, this study has clarified the interconnected pathological mechanisms underlying visual dysfunction in the COH rat model. However, several important limitations should be acknowledged. First, the COH rat model induces a progressively worsening injury. In this study, LbGp was administered immediately after modeling; therefore, its observed effects may reflect a dual action—both preventing injury progression and promoting functional repair. Future studies could distinguish between these two mechanisms by delaying LbGp administration after injury or by administering the compound at different time points. Second, measurements of visual function, retinal structure, retinal microcirculation, and related molecular mechanisms were performed only at the endpoint (day 28 post-modeling), without monitoring dynamic changes during the intervention period. As a result, the temporal evolution of each indicator remains unclear. Future research should include measurements at multiple time points to more comprehensively evaluate the effects of LbGp. Third, there is currently no direct evidence regarding the neuroprotective effects of LbGp in humans, as all findings are derived from animal models and *in vitro* experiments. Therefore, the translation of these results to human populations should be approached with caution. Well-designed clinical trials are needed in the future to assess the safety and efficacy of LbGp in human retinal diseases.

## 5 Conclusion

This study shows that LbGp effectively protects visual function, reduces retinal structural damage, and improves retinal microcirculation in COH rats. These neuroprotective effects are closely linked to the inhibition of the caspase-1/N-GSDMD/IL-1β-mediated pyroptosis pathway. The findings not only provide a new perspective on the pathophysiology of chronic intraocular pressure elevation, but also offer important experimental support and a promising therapeutic candidate for pyroptosis-targeted neuroprotection in glaucoma. Further research is needed to clarify the downstream targets and signaling networks involved in the regulation of pyroptosis.

## Data Availability

The original contributions presented in the study are included in the article/supplementary material, further inquiries can be directed to the corresponding authors.
